# Accuracy of continuous photoplethysmography-based 1 min mean heart rate assessment during atrial fibrillation

**DOI:** 10.1093/europace/euad011

**Published:** 2023-02-07

**Authors:** Astrid N L Hermans, Jonas L Isaksen*, Monika Gawalko, Nikki A H A Pluymaekers, Rachel M J van der Velden, Hilco Snippe, Stijn Evens, Glenn De Witte, Justin G L M Luermans, Martin Manninger, Joost Lumens, Jørgen K Kanters, Dominik Linz

**Affiliations:** Department of Cardiology, Maastricht University Medical Centre and Cardiovascular Research Institute Maastricht, P. Debyelaan 25, 6229 HX Maastricht, The Netherlands; Department of Biomedical Sciences, Faculty of Health and Medical Sciences, University of Copenhagen, Nørregade 10, 1165 Copenhagen, Denmark; Department of Cardiology, Maastricht University Medical Centre and Cardiovascular Research Institute Maastricht, P. Debyelaan 25, 6229 HX Maastricht, The Netherlands; Institute of Pharmacology, West German Heart and Vascular Centre, University Duisburg-Essen, Forsthausweg 2, 47057 Duisburg, Germany; 1st Department of Cardiology, Medical University of Warsaw, Żwirki i Wigury 61, 02-091 Warsaw, Poland; Department of Cardiology, Maastricht University Medical Centre and Cardiovascular Research Institute Maastricht, P. Debyelaan 25, 6229 HX Maastricht, The Netherlands; Department of Cardiology, Maastricht University Medical Centre and Cardiovascular Research Institute Maastricht, P. Debyelaan 25, 6229 HX Maastricht, The Netherlands; Department of Cardiology, Maastricht University Medical Centre and Cardiovascular Research Institute Maastricht, P. Debyelaan 25, 6229 HX Maastricht, The Netherlands; Qompium NV, Kempische Steenweg 293/16, 3500 Hasselt, Belgium; Qompium NV, Kempische Steenweg 293/16, 3500 Hasselt, Belgium; Department of Cardiology, Maastricht University Medical Centre and Cardiovascular Research Institute Maastricht, P. Debyelaan 25, 6229 HX Maastricht, The Netherlands; Department of Cardiology, Radboud University Medical Centre, Geert Grooteplein Zuid 10, 6525 GA Nijmegen, The Netherlands; Division of Cardiology, Department of Internal Medicine, Medical University of Graz, Auenbruggerpl. 2, 8036 Graz, Austria; Department of Biomedical Engineering, Cardiovascular Research Institute Maastricht (CARIM), Maastricht University, Minderbroedersberg 4-6, 6211 LK Maastricht, The Netherlands; Department of Biomedical Sciences, Faculty of Health and Medical Sciences, University of Copenhagen, Nørregade 10, 1165 Copenhagen, Denmark; Department of Cardiology, Maastricht University Medical Centre and Cardiovascular Research Institute Maastricht, P. Debyelaan 25, 6229 HX Maastricht, The Netherlands; Department of Biomedical Sciences, Faculty of Health and Medical Sciences, University of Copenhagen, Nørregade 10, 1165 Copenhagen, Denmark; Department of Cardiology, Radboud University Medical Centre, Geert Grooteplein Zuid 10, 6525 GA Nijmegen, The Netherlands; Centre for Heart Rhythm Disorders, University of Adelaide and Royal Adelaide Hospital, Port Rd, Adelaide SA 5000, Australia

**Keywords:** Persistent atrial fibrillation, 1 min mean heart rate assessment, Photoplethysmography, Electrocardiography, Mobile health

## Abstract

**Aims:**

Although mobile health tools using photoplethysmography (PPG) technology have been validated for the detection of atrial fibrillation (AF), their utility for heart rate assessment during AF remains unclear. Therefore, we aimed to evaluate the accuracy of continuous PPG-based 1 min mean heart rate assessment during AF.

**Methods and results:**

Persistent AF patients were provided with Holter electrocardiography (ECG) (for ≥24 h) simultaneously with a PPG-equipped smartwatch. Both the PPG-based smartwatch and Holter ECG automatically and continuously monitored patients’ heart rate/rhythm. ECG and PPG recordings were synchronized and divided into 1 min segments, from which a PPG-based and an ECG-based average heart rate estimation were extracted. In total, 47 661 simultaneous ECG and PPG 1 min heart rate segments were analysed in 50 patients (34% women, age 73 ± 8 years). The agreement between ECG-determined and PPG-determined 1 min mean heart rate was high [root mean squared error (RMSE): 4.7 bpm]. The 1 min mean heart rate estimated using PPG was accurate within ±10% in 93.7% of the corresponding ECG-derived 1 min mean heart rate segments. PPG-based 1 min mean heart rate estimation was more often accurate during night-time (97%) than day-time (91%, *P* < 0.001) and during low levels (96%) compared to high levels of motion (92%, *P* < 0.001). A neural network with a 10 min history of the recording did not further improve the PPG-based 1 min mean heart rate assessment [RMSE: 4.4 (95% confidence interval: 3.5–5.2 bpm)]. Only chronic heart failure was associated with a lower agreement between ECG-derived and PPG-derived 1 min mean heart rates (*P* = 0.040).

**Conclusion:**

During persistent AF, continuous PPG-based 1 min mean heart rate assessment is feasible in 60% of the analysed period and shows high accuracy compared with Holter ECG for heart rates <110 bpm.

## Graphical Abstract

**Graphical Abstract:**
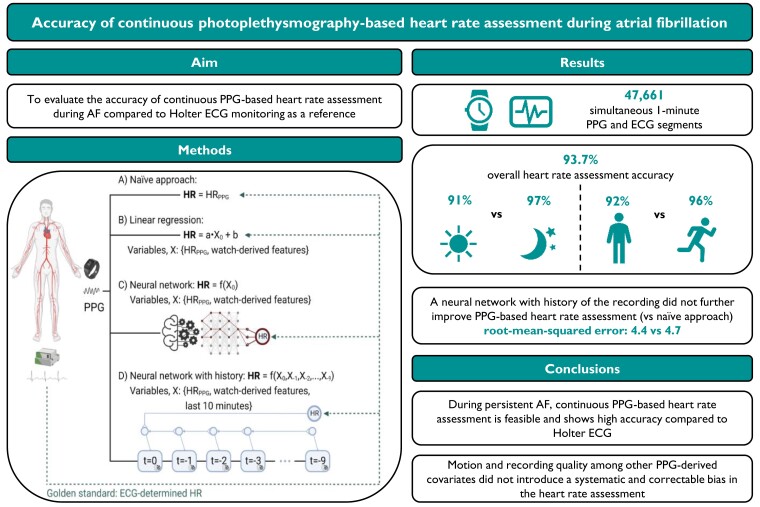

**What’s new?**

- Continuous photoplethysmography-based 1 min mean heart rate assessment during atrial fibrillation was feasible in 60% of the analysed period and showed high accuracy compared with Holter electrocardiography for heart rates <110 bpm.
- Photoplethysmography-based 1 min mean heart rate estimation was more often accurate during night-time than day-time and during low levels compared with high levels of motion.
- Motion and recording quality among other photoplethysmography-derived covariates did not introduce a systematic and correctable bias in the 1 min mean heart rate assessment.
- Patients with chronic heart failure more often had a lower agreement between electrocardiography-derived and photoplethysmography-derived 1 min mean heart rates.

## Introduction

According to the current European Society of Cardiology (ESC) guidelines, rate control is an integral part of atrial fibrillation (AF) management^1^ in order to improve AF-related symptoms.^2^ To date, lenient rate control (resting heart rate target <110 bpm) is recommended, unless a patient is highly symptomatic and requires a stricter rate control.^1,3^ To determine the adequacy of rate control, heart rate assessment based on scheduled or symptom-initiated electrocardiogram (ECG) monitoring tools, such as a 10 s resting ECG, Holter ECG, or event recorder is recommended.^4^ However, these heart rate monitoring tools can be costly and often cumbersome for patients in daily use. Therefore, mobile health (mHealth) solutions have been developed to overcome these limitations.^5–8^ Within the TeleCheck-AF project,^9^ a remote pathway consisting of photoplethysmography (PPG)-based heart rate/rhythm monitoring and teleconsultation has been created and introduced permanently to the standard of care for comprehensive AF management. Although mHealth tools using PPG technology have been validated for the detection of AF, it is unclear whether mHealth tools can accurately determine the heart rate during AF.^10^

In this prospective observational study, we aimed to (i) evaluate the accuracy of continuous PPG-based 1 min mean heart rate assessment during AF compared with Holter ECG monitoring as a reference and (ii) establish predictors for an accurate PPG-based 1 min mean heart rate assessment in patients with persistent AF.

## Methods

### Study population

From October 2020 to July 2021, consecutive patients (≥18 years) with persistent AF scheduled for Holter monitoring (of minimum 24 h) in the Maastricht University Medical Centre + (MUMC+), Maastricht, The Netherlands, were included. Individuals with implantable pacemakers were excluded.

### Study design

This prospective observational cohort study was performed in compliance with the Declaration of Helsinki and approved by the Institutional Review Board at the MUMC + (Committee reference number: NL 174232). All patients provided written informed consent.

### Study procedures

Outpatient clinic visits included Holter monitoring (minimum 24 h duration) (SEER Light monitor, GE Healthcare, Milwaukee, WI, USA) as a part of standard care. The Holter monitor provided three channels of diagnostic-quality ECG and its sampling rate was 128 samples per second. At this time point, patients were provided a PPG-equipped smartwatch (Samsung Galaxy Watch3, Samsung, Suwon, South Korea). The smartwatch software was Food and Drug Administration (FDA)-approved and Conformité Européenne (CE)-marked with a 25 Hz sampling rate. The manufacturer’s claim about the minimum heart rate that can be accurately measured using PPG was 20 bpm. There is no claim about the maximum heart rate that can be accurately measured. Patients were instructed to wear the smartwatch during the period of Holter monitoring. Both the PPG-equipped smartwatch and Holter automatically and continuously monitored patients’ heart rates and rhythm around the clock. All activities of daily living were allowed without any specific restrictions.

### Data collection and data analysis

Continuous recordings of ECG and PPG were aligned using time stamps and divided into separate 1 min segments. The PPG signals were preprocessed to remove noise and facilitate heartbeat detection. This process included interpolation at 30 Hz, a moving average filter, and a Savitzky–Golay filter, taking the derivative and normalizing the signal. Every channel of the raw PPG signal was preprocessed accordingly. The deep neural network of FibriCheck intrinsically calculated and extracted features from the preprocessed PPG signal and calculated the average 1 min heart rate based on these features. The covariates included (i) heart rate variability, (ii) a PPG signal quality metric, (iii) a motion index, and (iv) a variation in motion index. Heart rate variability was quantified as the root mean square of successive differences (RMSSDs) of detected heart beats. To categorize the PPG recordings into sufficient or insufficient quality to detect and differentiate heartbeats (quality metric), raw signals were analysed by a recurrent neural network algorithm of FibriCheck as described elsewhere.^11^ This deep neural network automatically determined the quality of the signal and annotated the signal accordingly. The network was trained to identify segments of insufficient signal quality based on the amount of noise. Heartbeats just within the sufficient signal quality segment of the 1 min PPG recording were used to compute the heart rate. The heart rate (beats per minute) was computed as 60 (seconds per minute), divided by the average of at least 20 beat-to-beat intervals (in seconds). The complete PPG recording was classified as insufficient quality when less than 20 heartbeats with sufficient quality were detected; for those PPG recordings with insufficient quality, PPG-based 1 min mean heart rate could not be assessed by the FibriCheck algorithm. Examples of PPG-based 1 min mean heart rate segments with insufficient and sufficient quality are presented in Supplementary material online, *Figure S1*. Motion was defined as the average magnitude of the accelerometer vector during the measurement. The accelerometer acquired 50 Hz signals. The motion index was determined for each measurement and recordings were classified based on quartiles (G1, G2, G3, or G4) after excluding recordings with insufficient quality. Variation in motion index was defined as the standard deviation (SD) of the motion (vector) over the entire measurement.

Holter recordings and annotations were exported from the MARS ambulatory Holter ECG analysis system (GE Healthcare, Milwaukee, WI, USA) and reviewed by a certified Holter ECG technician. The ECG-derived 1 min mean heart rate served as the golden standard. Simultaneous PPG and ECG 1 min segments with insufficient quality for analysis were excluded.

### Statistical analyses

The segments were categorized based on the ECG-based 1 min mean heart rate into three rate control ranges: ≤ 80 bpm, 80–110 bpm, and insufficient rate control >110 bpm.^3^ We defined an accurate PPG-based assessment of the 1 min mean heart rate as one that deviated less than ±10% from the corresponding ECG-derived 1 min mean heart rate. To evaluate the effect of time of recording on the accuracy of the PPG-based 1 min mean heart rate assessment, we split the data into day-time (predominantly active and/or awake period between 8 a.m. and 10 p.m.) and night-time (predominantly rest and/or sleeping period between 12 a.m. and 6 a.m.). Because people go to bed and get out of bed at different times, we excluded segments recorded between 10 p.m. and 12 a.m. and between 6 a.m. and 8 a.m., respectively, from the day-time/night-time analysis. To investigate the effect of motion on the accuracy of the 1 min mean heart rate assessment, we categorized the 1 min segments into four groups (G1–4) using the motion index. G1 was defined as minimum to lower quartile (≤9.85 m/s^2^), G2 as lower quartile to median (9.86–9.92 m/s^2^), G3 as median to upper quartile (9.93–10.04 m/s^2^), and G4 as upper quartile to maximum (≥10.05 m/s^2^; G4).

All continuous variables were tested for normality with the Shapiro–Wilk test. Variables with a normal distribution were presented as mean ± SD. Non-normal variables were expressed as median [interquartile range (IQR)] and categorical variables as numbers (*n*) with percentages (%). For the comparison of categorical data, Fisher’s exact tests were used. Differences in continuous parameters were compared using independent-samples *t*-tests and non-parametric Mann–Whitney *U* tests, as appropriate. The agreement between the PPG-based 1 min mean heart rate estimation and ECG-derived 1 min mean heart rate during AF was assessed using Bland–Altman analysis. We used the root mean squared error (RMSE) to quantify the disagreement. The RMSE is particularly sensitive to outliers (i.e. clinically relevant mistakes) and was defined as follows: $RMSE=\sqrt{\frac{1}{N}\underset{N}{\sum }\;{\left(HR_{PPG}-HR_{ECG}\right)}^{2}}$.

**Figure 1:**
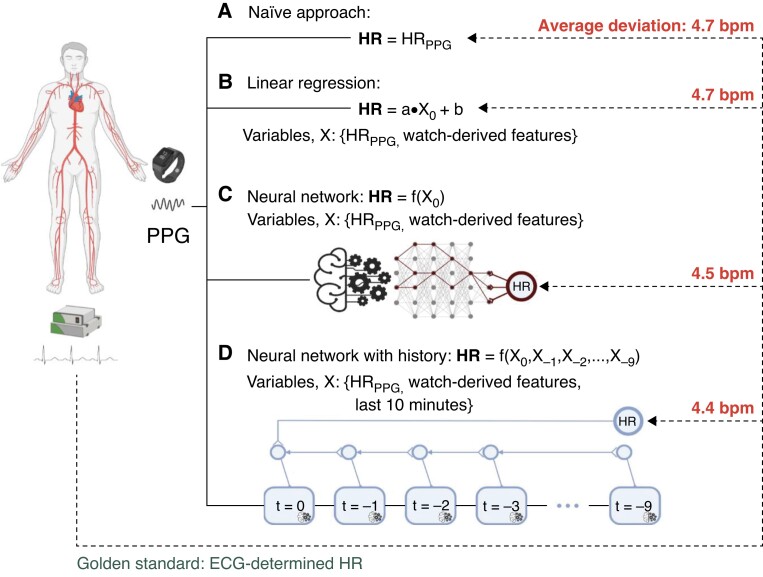
Four approaches to the 1 min mean heart rate estimation using PPG. The naïve approach (*A*) using the PPG-derived 1 min mean heart rate at face value demonstrates a low deviation from the ECG-determined 1 min mean heart rate. The linear regression (*B*) incorporates the watch-derived features of heart rate variability, motion, motion variability, and PPG signal quality, and demonstrates no significant systematic bias. The simple neural network (*C*) allows non-linear corrections for covariates, and the advanced neural network (*D*) has access to the nine preceding minutes of recording, in addition to the segment under analysis. The neural networks make insignificantly better predictions of the true 1 min mean heart rate than the raw PPG predictions, suggesting little to no systematic bias from heart rate variability, movement, and PPG signal quality on the PPG-based 1 min mean heart rate assessment. ECG, electrocardiography; HR, heart rate; PPG, photoplethysmography.

We took four approaches for the PPG-based 1 min mean heart rate estimation (*Figure 1*). The naïve approach (A) amounted to taking the PPG-based estimate at face value. To adjust for a possible systematic bias or trend, we employed a linear regression model (B). Because some biases may work in a non-linear way (e.g. only at higher heart rates or high levels of noise), we employed a simple neural network (C) to correct any such biases. This model C had two hidden layers with ten neurons each. Sudden changes in the PPG-based heart rate might conceivably be an incorrect measurement due to movement or noise, and thus, we constructed an advanced neural network (D) which was supplied with data from up to nine previous minutes along with data from the segment under analysis. The duration of recording history was arbitrarily chosen to give a proper baseline. Model D was designed with memory using blocks of long short-term memory (LSTM).^12^ The network consisted of two parallel processing paths each with four LSTM blocks with five units each, followed by a fully connected layer with eight neurons. The two paths were concatenated at the fully connected level and connected to one final output neuron.

Models B–D were fitted and tested using 5-fold cross-validation to make the approaches comparable. Model A required no fitting of parameters, making cross-validation obsolete for this model. A *P*-value ≤ 0.05 was considered statistically significant. We used IBM SPSS Version 28 (IBM Corporation, Somers, New York, USA) for database management and statistical analysis, and models were fitted using Tensorflow v. 2.8 in Python v. 3.8.

## Results

We included 50 consecutive patients with persistent AF (34% women, age 73 ± 8 years) in the present study and divided those in patients with a high (≥95%) or low (<95%) agreement rate between the PPG-based 1 min mean heart rate assessment and ECG-derived 1 min mean heart rate assessment (*Table 1*). Many (70%) had hypertension and 26% had chronic heart failure. Most patients (88%) had an increased thromboembolic risk (CHA_2_DS_2_-VASc score ≥2 in men or ≥3 in women), and 96% were anticoagulated. The duration of the Holter recording was 24 h in 45 patients (90%) and 48 h in 5 patients (10%).

**Table 1:** Clinical characteristics of included patients, divided into two groups according to the agreement rate between PPG and ECG heart rates

Variable	Study group (*n* = 50)	Low agreement rate (*n* = 15)	High agreement rate (*n* = 35)	*P-*value
**Demographics**				
Age (years) – median (IQR)	75 (68–79)	75 (62–82)	75 (69–78)	0.840
Female sex	17 (34%)	3 (20%)	14 (40%)	0.171
BMI (kg/m^2^) – median (IQR)	27.6 (24.7–31.9)	27.5 (24.7–29.4)	27.8 (24.6–32.1)	0.582
**AF**				
First-detected AF	5 (10%)	1 (7%)	4 (11%)	1.000
Previous CV (electrical and/or pharmacological) ^a^	20/45 (44%)	5/14 (36%)	15/31 (48%)	0.428
Ablation therapy for AF^a^	4/45 (9%)	1/14 (7%)	3/31 (10%)	1.000
**Cardiovascular diseases**				
Myocardial infarction	9 (18%)	5 (33%)	4 (11%)	0.106
PCI/PTCA	6 (12%)	3 (20%)	3 (9%)	0.348
CABG	4 (8%)	1 (7%)	3 (9%)	1.000
Peripheral vascular disease	2 (4%)	0 (0%)	2 (6%)	1.000
Diabetes mellitus	7 (14%)	1 (7%)	6 (17%)	0.659
Hypertension	35 (70%)	9 (60%)	26 (74%)	0.333
Chronic heart failure	13 (26%)	7 (47%)	6 (17%)	0.040
Obesity (BMI ≥30 kg/m2)	15 (30%)	2 (13%)	13 (37%)	0.176
Stroke/TIA/pulmonary embolism	11 (22%)	3 (20%)	8 (23%)	1.000
**Thromboembolic risk**				
CHA_2_DS_2_-VASc score = 0 (if male), = 1 (if women)	2 (4%)	1 (7%)	1 (3%)	0.514
CHA_2_DS_2_-VASc score ≥2 (if male), ≥ 3 (if women)	44 (88%)	12 (80%)	32 (91%)	0.348
**Medication**				
Oral anticoagulants	48 (96%)	15 (100%)	33 (94%)	1.000
Antiplatelet drugs	3 (6%)	1 (7%)	2 (6%)	1.000
Beta-blockers	39 (78%)	11 (73%)	28 (80%)	0.713
Antiarrhythmic drugs	4 (8%)	2 (13%)	2 (6%)	0.574
Diuretics	20 (40%)	7 (47%)	13 (37%)	0.529
CCB	13 (26%)	4 (27%)	9 (26%)	1.000
ACEI	15 (30%)	5 (33%)	10 (29%)	0.747
ARB	16 (32%)	7 (47%)	9 (26%)	0.191
MRA	4 (8%)	1 (7%)	3 (9%)	1.000
Digoxin	11 (22%)	6 (40%)	5 (14%)	0.070

Values are depicted as the number of patients (*n*) with percentages unless indicated otherwise.

Results after excluding patients with first-detected AF.

ACEI, angiotensin-converting enzyme inhibitor; AF, atrial fibrillation; ARB, angiotensin receptor blocker; BMI, body mass index; CABG, coronary artery bypass surgery; CCB, calcium channel blockers; CRT, cardiac resynchronization therapy; CV, cardioversion; ICD, implantable cardioverter-defibrillator; IQR, interquartile range; MRA, mineralocorticoid receptor antagonists; PCI, percutaneous coronary intervention; PM, pacemaker; PTCA, percutaneous transluminal coronary angioplasty; TIA, transient ischaemic attack.

### PPG-based and ECG-based 1 min mean heart rate assessment

The recordings from 50 patients amounted to 79 443 min of simultaneous ECG- and PPG- recording, of which 47,661 1 min segments (60%) passed the inclusion criteria. A total of 31 782 (40%) simultaneous PPG and ECG segments with insufficient quality for analysis were excluded. Ninety segments were excluded for ECG quality insufficiency, 24 006 segments for PPG-based insufficient quality, and 7686 segments for both ECG and PPG quality insufficiency. Based on ECG analysis, 30 968 segments (65%) showed a 1 min mean heart rate ≤80 bpm of which 299 segments (1%) ≤ 40 bpm, 15 239 segments (32%) showed a 1 min mean heart rate between 80 and 110 bpm, and 1454 segments (3%) showed a 1 min mean heart rate of >110 bpm.

**Figure 2:**
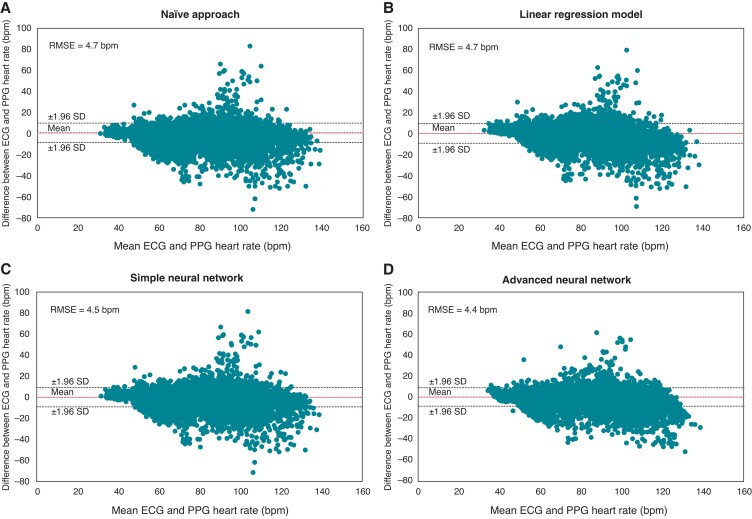
Agreement between the ECG-derived and PPG-based 1 min mean heart rate. Bland–Altman plots for the four approaches (see *Figure 1* and the text for a description of the methods). ECG, electrocardiography; PPG, photopletysmography; RMSE, root mean squared error; SD, standard deviation.

**Figure 3:**
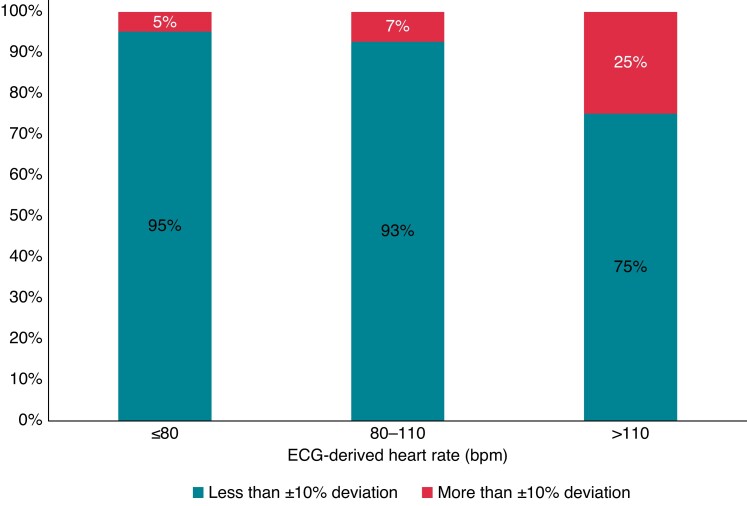
Percentage of PPG-based 1 min mean heart rate assessments that are accurate to within ±10%. ECG, electrocardiography; PPG, photopletysmography.

Across all simultaneous ECG and PPG recordings with sufficient quality, the mean, minimum, and maximum for ECG-derived 1 min mean heart rates were 75 ± 16 bpm, 30 bpm, and 157 bpm, respectively, and for PPG-based 1 min mean heart rates were74 ± 16 bpm, 31 bpm, and 167 bpm, respectively. Included covariates had a median of 0.26 (0.20–0.32) ms for RMSSD, 0.03 (0.00–0.22) for PPG signal quality, 9.90 (9.84–9.98) m/s^2^ for motion index, and 0.05 (0.04–0.28) m/s^2^ for motion index variation. Substituting the PPG-based 1 min mean heart rate for the ECG-derived 1 min mean heart rate is associated with an RMSE of 4.7 bpm and the Bland–Altman lower and upper limit boundaries (defined as ±1.96 SD) are −8.4 bpm and 9.9 bpm, respectively (*Figure 2A*). The linear regression, which would correct any systematic bias in the PPG-based 1 min mean heart rate estimation is associated with an error of 4.7 bpm and the limits of agreement are −9.2 to 9.5 bpm [95% CI: 3.9–5.5], indicating that no systematic bias is found (*Figure 2B*). The simple neural network, designed to correct any non-linear bias, is associated with an error of 4.5 bpm and the limits of agreement are −9.0 to 9.2 bpm [95% CI: 3.6–5.5], which is also not significantly better than the naïve approach (*Figure 2C*). We found no evidence of outliers in the PPG-based 1 min mean heart rate assessments that could be corrected with the use of a sliding window of 10 min [error: 4.4 bpm and limits of agreement: −8.4 to 9.1 bpm (95% CI: 3.5–5.2), *Figure 2D*]. The PPG-based 1 min mean heart rate estimates were accurate within ±10% in 93.7% of the 1 min segments (95% CI: 93.5–94.0%). Deviations >±10% were seen among all 50 patients; with a median number of PPG-based 1 min mean heart rate estimates with deviations >±10% per patient of 26 (16–46). Accuracy declined with an increased 1 min mean heart rate. Of the segments with a 1 min mean heart rate ≤80 bpm (65%), the PPG-based 1 min mean heart rate assessment deviated less than ±10% from the corresponding ECG-derived 1 min mean heart rate in 95%. For segments with an ECG-derived 1 min mean heart rate of 80–110 bpm (32%), accuracy was 93%. Of the segments with a 1 min mean heart rate >110 bpm (3%), the PPG-based 1 min mean heart rate assessment deviates less than ±10% from the corresponding ECG-derived 1 min mean heart rate in 75% (*Figure 3*). Even for very low 1 min mean heart rates (≤40 bpm) (1%), accuracy remained high at 92%.

### 1 min mean heart rate assessment during activity and during night-time

Of the 47 661 PPG 1 min mean heart rate fragments, 38 529 (81%) were available for day-time/night-time analysis and 9132 (19%) segments were excluded as they were recorded between 10 p.m. and 12 a.m. and between 6 a.m. and 8 a.m. A total of 22 176 simultaneous recordings (58%) are performed during day-time and 16 353 (42%) during night-time (*Table 2*). The PPG-based 1 min mean heart rate assessment is more accurate during night-time compared with day-time (15 811 (97%) vs. 20 162 (91%), *P* < 0.001) (*Table 4*). Correspondingly, categorizing recordings into the correct heart rate group (≤80, 80–110, > 110 bpm, i.e. a rough assessment) is more successful during night-time compared with day-time [15 956 (98%) vs. 20 058 (90%), *P* < 0.001] (*Table 2*).

**Table 2:** PPG-based 1 min mean heart rate and ECG-derived 1 min mean heart rate fragments during day-time and night-time

		PPG-based 1 min mean heart rate					
		Day			Night		
		≤80 bpm	80–110 bpm	>110 bpm	≤80 bpm	80–110 bpm	>110 bpm
**ECG-derived 1 min mean heart rate**	**≤80 bpm**	10 720 (93.5%)	722 (6.3%)	23 (0.2%)	13 422 (99.1%)	120 (0.9%)	1 (0.0%)
	**80–110 bpm**	836 (8.8%)	8593 (90.4%)	77 (0.8%)	256 (9.2%)	2514 (90.6%)	5 (0.2%)
	**>110 bpm**	9 (0.7%)	451 (37.4%)	745 (61.8%)	0	15 (43%)	20 (57%)

The green boxes represent the accurately assessed PPG-based 1 min mean heart rate fragments and the red boxes represent the inaccurately assessed PPG-based 1 min mean heart rate fragments when using the ECG-derived 1 min mean heart rate as reference.

ECG, electrocardiography; PPG, photoplethysmography.

**Table 4:** PPG-based 1 min mean heart rate estimation when considering day-time/night-time and motion levels

*PPG-based 1 min mean heart rate estimation when considering the following 1 min recordings:*	RMSE	Bland–Altman Lower – Upper limit boundary
Day-time	6.0	−10.7–12.8
Night-time	2.8	−5.0–5.9
Low motion (G1)	3.5	−6.4–7.5
Low-medium motion (G2)	4.5	−8.2–9.5
Medium-high motion (G3)	5.4	−9.7–11.5
High motion (G4)	5.6	−9.8–12.0

PPG, photoplethysmography; RMSE, root mean squared error.

Motion data were available for all 47,661 1 min segments. A total of 14 796 simultaneous recordings (31%) are categorized in the motion quartile G1, 14 321 (30%) in G2, 11 758 (25%) in G3, and 6786 (14%) in G4 (*Table 3*). The accuracy of the PPG-based 1 min mean heart rate assessment is higher during lower motion levels compared with the higher motion levels [G1: 14 149 (96%) vs. G2: 13 458 (94%) vs. G3: 10 806 (92%) vs. G4 6268 (92%), *P* < 0.001] (*Table 4*). Categorizing recordings into the right heart rate group (≤80 bpm vs. 80–110 bpm vs. >110 bpm) is more accurate at lower motion levels compared with higher motion levels [G1: 14 337 (97%) vs. G2: 13 422 (94%) vs. G3: 10 821 (92%) vs. G4: 6134 (90%), respectively, *P* < 0.001] (*Table 3*).

**Table 3:** PPG-based 1 min mean heart rate and ECG-derived 1 min mean heart rate fragments per motion-based quartile

		Low motion (G1)			Low-medium motion (G2)			Medium-high motion (G3)			High motion (G4)		
PPG-based 1 min mean heart rate		≤80 bpm	80–110 bpm	>110 bpm	≤80 bpm	80–110 bpm	>110 bpm	≤80 bpm	80–110 bpm	>110 bpm	≤80 bpm	80–110 bpm	>110 bpm
**ECG-derived 1 min mean heart rate**	**≤80 bpm**	11 537 (36%)	180 (1%)	7 (0.02%)	9,539 (30%)	294 (1%)	11 (0.03%)	6,796 (21%)	347 (1%)	3 (0.01%)	3,079 (10%)	202 (1%)	5 (0.02%)
	**80–110 bpm**	227 (2%)	2733 (19%)	6 (0.04%)	450 (3%)	3,688 (26%)	18 (0.13%)	383 (3%)	3,784 (26%)	36 (0.25%)	238 (2%)	2,757 (19%)	35 (0.24%)
	**>110 bpm**	0	39 (3%)	67 (5%)	2 (0.15%)	124 (9%)	195 (15%)	4 (0.31%)	164 (13%)	241 (18%)	3 (0.23%)	169 (13%)	298 (23%)

PPG 1 min mean heart rate fragments are split into motion index-based groups (G). G1 is defined as minimum to lower quartile (≤9.85 m/s^2^), G2 as lower quartile to median (9.86–9.92 m/s^2^), G3 as median to higher quartile (9.93–10.04 m/s^2^), and G4 as higher quartile to maximum (≥10.05 m/s^2^). The green boxes represent the accurately assessed PPG-based 1 min mean heart rate fragments and the red boxes represent the inaccurately assessed PPG-based 1 min mean heart rate fragments when using the ECG-derived 1 min mean heart rate as reference. *Abbreviations:* ECG, electrocardiography; PPG, photoplethysmography.

### Clinical predictors of poor PPG predictions

**Figure 4:**
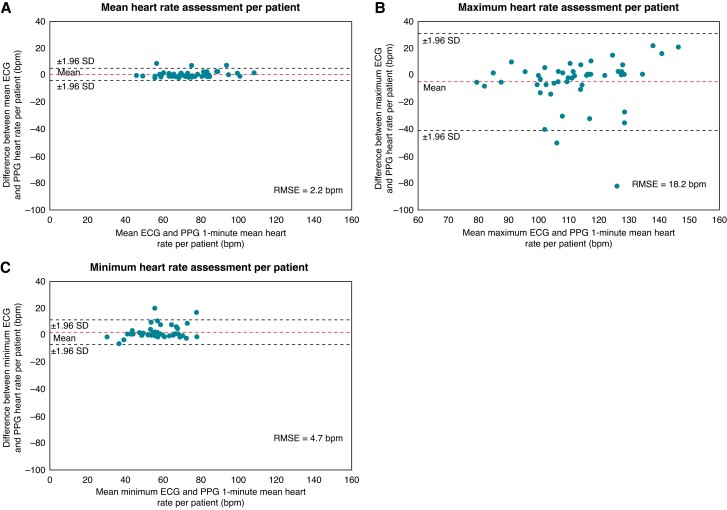
Agreement between the mean, maximum, and minimum ECG-derived and PPG-based 1 min mean heart rates per patient. ECG, electrocardiography; PPG, photoplethysmography; RMSE, root mean squared error; SD, standard deviation.

On average, 953 ± 259 1 min segments were available per patient. The mean 1 min mean heart rate per patient was 74 ± 13 bpm based on ECG and 73 ± 13 bpm based on PPG. Substituting the mean PPG-based 1 min mean heart rate for the mean ECG-derived 1 min mean heart rate per patient is associated with an RMSE of 2.2 bpm and the Bland–Altman lower and upper limit boundaries (defined as ±1.96 SD) are −3.6 bpm and 5.1 bpm, respectively (*Figure 4A*). The ECG-derived and PPG-based maximum 1 min mean heart rates per patient were 110 ± 18 bpm and 115 ± 16 bpm, respectively. Substituting the PPG-based maximum 1 min mean heart rate for the ECG-derived maximum 1 min mean heart rate per patient is associated with an RMSE of 18.2 bpm and the Bland–Altman lower and upper limit boundaries (defined as ±1.96 SD) are −40.6 bpm and 30.9 bpm, respectively (*Figure 4B*). The ECG-derived and PPG-based minimum 1 min mean heart rates per patient were 58 ± 11 bpm and 56 ± 10 bpm, respectively. Substituting the PPG-based minimum 1 min mean heart rate for the ECG-derived minimum 1 min mean heart rate per patient was associated with an RMSE of 4.7 bpm and the Bland–Altman lower and upper limit boundaries (defined as ±1.96 SD) are −6.9 bpm and 11.4 bpm, respectively (*Figure 4C*). The proportion of recording time per patient with an ECG-derived 1 min mean heart rate ≤110 bpm was 98 ± 7% and with a PPG-based 1 min mean heart rate ≤110 bpm was 98 ± 6%. Two thousand nine hundred and eighty (6.3%) PPG recordings deviated more and 44 681 (93.7%) PPG recordings deviated less than ±10% from the corresponding ECG-derived 1 min mean heart rate. The mean and median proportion of inaccurate measurements (deviation more than ±10%) per patient were 6.5%±11.4% and 2.9% (1.7–6.6), respectively. Most patients had a good agreement between the PPG-based 1 min mean heart rate assessment and ECG-derived 1 min mean heart rate assessment, but 15 patients (30%) had an agreement rate <95%. Detailed characteristics of patients with low and high agreement rates are presented in *Table 1*. Only chronic heart failure was more common in the group with a low agreement rate [7 (47%) vs. 6 (17%), *P* = 0.040].

## Discussion

The utility and limitations of PPG technology to assess heart rate during AF have been addressed and discussed in previous studies.^13–15^ This study is the first to evaluate the accuracy of continuous PPG-based 1 min mean heart rate monitoring in patients with persistent AF. The main findings of our study are as follows. First, PPG technology is suitable to accurately assess the 1 min mean heart rate during AF without a clinically significant bias compared with Holter ECG monitoring as a reference. Secondly, the accuracy of the PPG-based 1 min mean heart rate assessment was better during night-time than day-time and during lower motion levels compared with higher motion levels. Thirdly, a neural network with access to the PPG-derived 1 min mean heart rate and additional covariates did not augment the already high validity of the 1 min mean heart rate assessment. Finally, the agreement rate between the PPG-based 1 min mean heart rate and ECG-derived 1 min mean heart rate was lower in patients with a clinical history of chronic heart failure.

We found a strong agreement between the 1 min mean heart rate during AF assessed by PPG technology with simultaneous ECG recordings, which supports the use of PPG-based 1 min mean heart rate monitoring as a feasible strategy for rate control management in AF patients. The current ESC guidelines recommend a lenient rate control strategy with a resting heart rate below 110 bpm^1,3^ and identifying periods with too fast (tachyarrhythmia) or too slow (bradyarrhythmia) ventricular responses during AF is important to maintain optimal exercise tolerance and reduce the risk of heart failure (tachycardiomyopathy).^16^ Theoretically, underestimation of fast heart rates during AF can occur due to the so-called pulse deficit.^17^ The high beat-to-beat variability in AF may result in a variable diastolic filling of the ventricular system and, consequently, in reduced amplitudes of the PPG peaks due to changes in the perfusion of the microvasculature. This may impair signal recognition and heart rate computation in the PPG waveforms, which makes the estimation of heart rate from peripheral pulse more challenging, especially at higher heart rates.^13,18^ In our study, 3% of the 1 min ECG fragments showed a mean heart rate above 110 bpm, and in 75% of those cases, the PPG-based 1 min mean heart rate assessment deviated less than ±10% from the corresponding ECG-derived 1 min mean heart rate. Furthermore, only 1% of the 1 min ECG fragments showed a 1 min mean heart rate ≤40 bpm, and in 92% of those cases, the PPG-based 1 min mean heart rate assessment deviated less than ±10% from the corresponding ECG-derived 1 min mean heart rate. Thus, the PPG-based 1 min mean heart rate estimation seems feasible to guide lenient rate control during AF. However, in some cases, PPG may underestimate true faster 1 min mean heart rates >110 bpm during AF. The best way to deal with this problem clinically remains uncertain. Whether novel PPG algorithms incorporating additional PPG waveform features identifying possible pulse loss and consequent underestimation of heart rate or additional ECG recordings represent the best solution remains to be determined. Additionally, it is important to note that despite a good performance of PPG to assess slow 1 min mean heart rates during AF, the mechanism of heart rates ≤40 bpm (e.g. AV block or sinus arrest) cannot be assessed based on PPG recordings.^14^

Despite being overall effective in assessing the 1 min mean heart rate, our results showed that the accuracy of the 1 min mean heart rate assessment by PPG technology may be limited by some factors impacting the recording quality. Patient movement has been shown previously to lead to motion artefacts in PPG signals, which negatively impacts the proportion of insufficient quality PPG recording fragments.^19^ In our analysis, we confirmed this finding and showed that after the exclusion of insufficient quality fragments, motion did still affect the accuracy of the PPG-based 1 min mean heart rate estimation. We also observed that the PPG-based 1 min mean heart rate assessment during night-time was more accurate than day-time. Factors such as a lower degree of respiratory arrhythmia and the absence of various heart rate stimuli during the night may partially explain this finding.^20^ Therefore, periods of low physical activity and the night-time provide an excellent time window for the PPG-based 1 min mean heart rate assessment during AF. As suggested in previous studies, further integration of accelerometer information in the PPG sensor could provide a means of quantifying the displacement of the sensor during use and may help to correct for possible movement artefacts in PPG signals.^21^ Despite the high validity of 1 min mean heart rate assessment using the crude PPG measurements, we sought to further improve accuracy with the use of artificial intelligence. PPG signals are well-suited data for machine learning approaches and have been used primarily in deep neural networks to detect AF.^22,23^ Although we saw a trend toward more accurate 1 min mean heart rate estimations when motion index and recording quality among other covariates were supplied to a deep learning model, we saw no significant improvement. Importantly, all inputs were post-processed data (e.g. the PPG-derived heart rate of a 60 s segment), and it is possible that the application of deep learning to the waveforms derived from a raw PPG signal may yield higher accuracy and diminish the lack of beat recognition due to the pulse deficit problem.^17,24^ A 9 min history did not lead to a significantly more accurate 1 min mean heart rate prediction despite the trend to improvement. We speculate that outliers/extreme errors, which the history might help to identify, are uncommon or may have already been excluded due to poor recording quality. These findings indicate that crude mHealth PPG-based 1 min mean heart rate assessment, which is feasible and robust, does not require further artificial intelligence augmentation.

Furthermore, patients with a high agreement rate between the PPG-based 1 min mean heart rate and ECG-derived 1 min mean heart rate less frequently had chronic heart failure compared with those with a low agreement rate. The exact reason remains unclear, but factors such as arterial stiffness and progressed atherosclerosis have been shown to impact the shape and timing of the PPG pulse wave,^25^ which may contribute to the observed low agreement rate between the ECG-derived and PPG-based heart rate in patients with chronic heart failure. Whether chronic heart failure should stop us from using PPG technology for the assessment of heart rate during AF warrants further prospective evaluation. Furthermore, although we found no association between chronic heart failure and covariates such as the use of beta-blockers, ECG-derived 1 min mean heart rate as well as the level of motion (see Supplementary material online, *Table S1*), we cannot exclude the influence of these covariates in patients with chronic heart failure on the accuracy of the PPG-based 1 min mean heart rate assessment due to the small size of the study group.

### Limitations

Our study has several limitations. First, although we had a large number of combined PPG and ECG recording segments available to evaluate the accuracy of the PPG-based 1 min mean heart rate estimation, the number of recruited patients was limited which may have impaired the identification of predictors for an accurate 1 min mean heart rate assessment during AF. Secondly, we included only persistent AF patients and it remains unclear whether the findings can be generalized to all patients with AF. Data recorded during sinus rhythm and the transition of the heart rhythm (from AF to sinus rhythm or vice versa) would be needed to evaluate how the PPG-based 1 min mean heart rate assessment performs in paroxysmal AF. Thirdly, we examined the accuracy of the PPG-based 1 min mean heart rate assessment during 24 h of monitoring, but further long-term follow-up recordings are needed to evaluate the performance during longer recordings. Fourth, due to the combined use of one specific software algorithm and one specific smartwatch device, the results cannot be generalized to other vendors and models without validation. Fifth, 40% of the 1 min segments did not pass the inclusion criteria for sufficient quality, which might have impacted the results. However, we showed that the PPG-based 1 min mean heart rate assessment gives feasible heart rate estimations to guide lenient rate control despite a high number of missing 1 min PPG recordings. Sixth, the number of ECG-derived 1 min mean heart rate fragments >110 bpm was limited. Therefore, capability of the PPG-based 1 min mean heart rate estimation at higher rates remains uncertain. Despite that, in 75% of the 1454 cases with a 1 min mean heart rate >110 bpm, the PPG-based 1 min mean heart rate assessment deviated less than ±10% from the corresponding ECG-derived 1 min mean heart rate. Seventh, the Bland–Altman analysis only defined the interval of agreement between the ECG-derived and PPG-based 1 min mean heart rate assessment; it does not say whether those limits are acceptable or not as this depends on clinical necessity. Finally, we assessed the mean heart rate computed over 1 min segments instead of a beat-to-beat heart rate for the determination of the PPG-based heart rate.

## Conclusions

Continuous PPG-based 1 min mean heart rate assessment during AF seems feasible to guide a lenient rate control and shows good accuracy compared with Holter ECG as a reference. Future studies need to be performed to evaluate how to integrate PPG-derived heart rate information into clinical decision-making processes to guide rate control in patients with AF. Motion and recording quality among other PPG-derived covariates did not introduce a systematic and correctable bias in the 1 min mean heart rate assessment. Chronic heart failure was associated with lower accuracy of the PPG-based 1 min mean heart rate assessment.

## Supplementary Material

euad011_Supplementary_Data

## Data Availability

The data underlying this article will be shared on reasonable request to the corresponding author.
