# Supplementary material for: Accuracy of continuous photoplethysmography-based 1 min mean heart rate assessment during atrial fibrillation
Source: Europace. 2023 Feb 7;25(3):835–44. doi: 10.1093/europace/euad011 (PMC10062358; doi:10.1093/europace/euad011)
Supplement: euad011_Supplementary_Data [file euad011_supplementary_data.docx]

| **Variable** | **Chronic heart failure** | | |
| --- | --- | --- | --- |
|  | **No (n=37)** | **Yes (n=13)** | ***P-*value** |
| Beta-blockers – n (%) | 28 (76%) | 11 (85%) | 0.704 |
| ECG-derived heart rate (bpm) – median (IQR) | 73 (64-82) | 78 (63-83) | 0.536 |
| Level of motion (m/s²) – median (IQR) | 9.90 (9.87-9.94) | 9.90 (9.87-9.92) | 0.870 |

**Table S1**. Covariates for accurate PPG-based heart rate assessment per patient based on chronic heart failure.

*Abbreviations:* bpm, beats per minute; IQR, interquartile range; PPG, photoplethysmography

**Figure S1**. Examples of 1-minute PPG-based heart rate recordings with (in)sufficient quality.
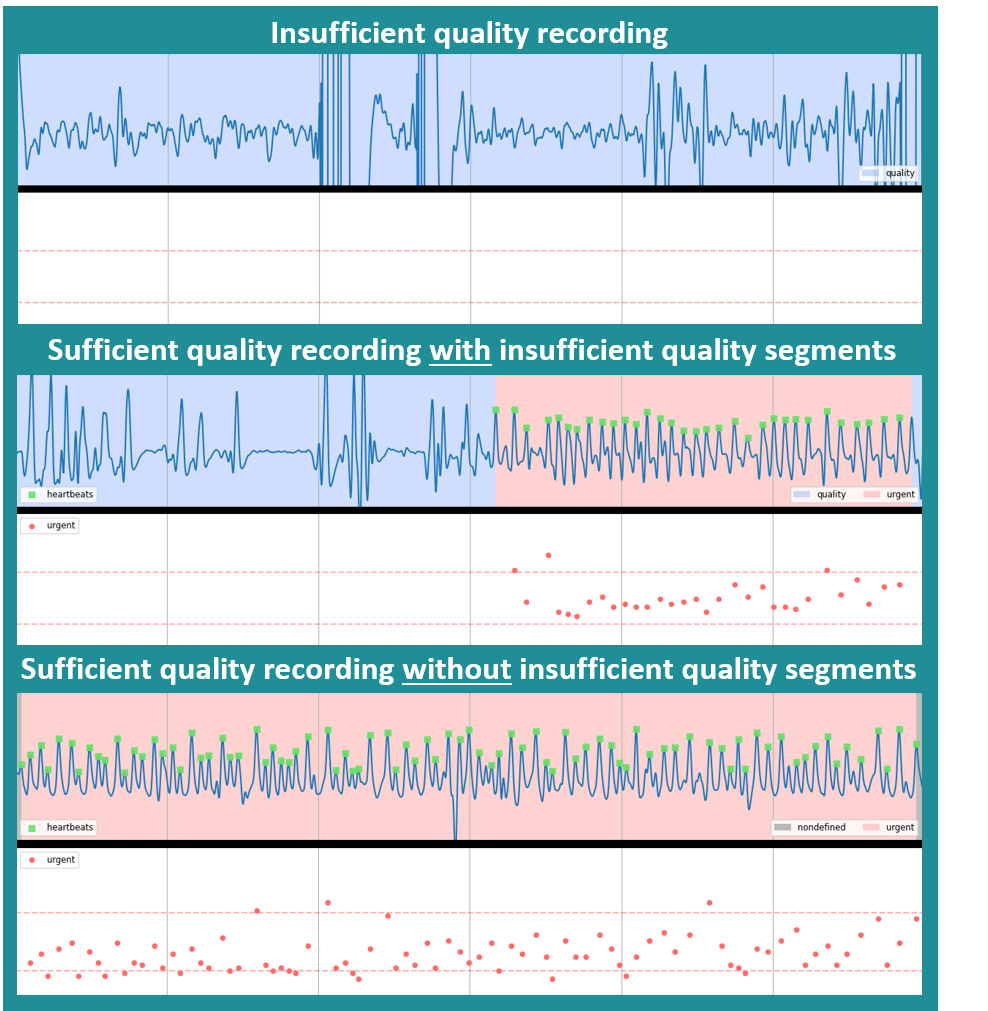

The *upper* example represents a PPG-based heart rate recording with insufficient quality (less than 20 heart beats with sufficient quality were detected). Such recording was excluded from analysis in its entirety. The *middle* example represents a PPG-based heart rate recording with sufficient quality (20 or more heart beats with sufficient quality were detected) but with several insufficient quality segments included. Only the sufficient quality segments of this recording were included in analysis. The *lower* example represents a PPG-based heart rate recording with sufficient quality (all detected heart beats were of sufficient quality). Such recording was included in analysis in its entirety. Red dots indicate heart beats with sufficient quality accurately detected by the algorithm.
*Abbreviations*: PPG, photoplethysmography
